# Supplementary material for: A Diagnostic Algorithm Based on a Simple Clinical Prediction Rule for the Diagnosis of Cranial Giant Cell Arteritis
Source: J Clin Med. 2021 Mar 10;10(6):1163. doi: 10.3390/jcm10061163 (PMC8001831; doi:10.3390/jcm10061163)
Supplement: Supplementary file 1 [file jcm-10-01163-s001.pdf]

Journal of
Clinical Medicine

Table S1: Final clinical diagnoses other than cGCA in cohort 1 (n = 61).

Patient	Sex	Age	Final clinical diagnosis
C1_a01	Female	56	Isolated extracranial GCA
C1_a02	Female	66	Isolated extracranial GCA
C1_a03	Male	66	Antiphospholipid-syndrome
C1_a04	Female	73	Noninflammatory arthalgia and myalgia
C1_a05	Female	85	Polymyalgia rheumatica, left heart failure
C1_a06	Female	60	Late onset rheumatoid arthritis
C1_a07	Female	50	Optic neuritis
C1_a08	Female	64	Polymyalgia rheumatica
C1_a09	Female	79	Hyporegenerative anemia
C1_a10	Male	58	Crohn`s disease with arthritis
C1_a11	Female	66	Anterior ischemic optic neuropathy, non arteritic
C1_a12	Female	53	Bacterial infection with unknown focus
C1_a13	Male	82	Central retinal artery occlusion, embolic
C1_a14	Male	77	Central retinal artery occlusion, embolic[§]
C1_a15	Female	69	Polymyalgia rheumatica
C1_a16	Female	51	Overlap polymyalgia rheumatica/rheumatoid arthritis
C1_a17	Female	90	Benign paroxysmal positional vertigo
C1_a18	Male	50	Aortic dissection, non arteritic
C1_a19	Female	57	Drug induced myalgia, arthralgia
C1_a20	Male	81	Fever of unknown origin, final diagnosis myelodysplastic syndrome
C1_a21	Female	71	Bacterial infection with unknown focus
C1_a22	Male	69	Overlap polymyalgia rheumatica/late onset rheumatoid arthritis
C1_a23	Female	78	Transient ischemic attack, non arteritic
C1_a24	Female	50	Migraine; anterior ischemic optic neuropathy, non arteritic
C1_a25	Female	61	Reactive arthritis
C1_a26	Male	53	Amaurosis fugax, embolic
C1_a27	Female	57	Isolated extracranial GCA
C1_a28	Female	76	Cervical spine syndrome, advanced arteriosclerosis oft he supraaortic arteries
C1_a29	Male	59	Isolated extracranial GCA
C1_a30	Male	72	Arthritis; Fever of unknown origin
C1_a31	Female	50	Atypical facial pain
C1_a32	Female	54	Alzheimer`s disease
C1_a33	Male	51	Sarcoidosis
C1_a34	Male	61	Abdominal periaortitis
C1_a35	Female	57	Polymyalgia rheumatica
C1_a36	Female	69	Central retinal artery occlusion, embolic
C1_a37	Male	53	Branch retinal artery occlusion, embolic
C1_a38	Female	72	Isolated extracranial GCA
C1_a39	Male	65	Transient ischemic attack (posterior circulation)
C1_a40	Female	66	Isolated extracranial GCA
C1_a41	Male	78	Noninflammatory arthalgia and myalgia
C1_a42	Male	66	ANCA-associated vasculitis
C1_a43	Female	67	Central retinal artery occlusion, embolic
C1_a44	Male	67	Hypertensive crisis with central retinal artery/vein occlusion
C1_a45	Female	65	Transient headache[§]
C1_a46	Female	52	ANCA-associated vasculitis
C1_a47	Male	82	Anterior ischemic optic neuropathy, non arteritic[§]
C1_a48	Male	75	Late onset rheumatoid arthritis
C1_a49	Male	76	Anterior ischemic optic neuropathy, non arteritic

C1_a50	Male	59	Fibromyalgia
C1_a51	Male	74	Anterior ischemic optic neuropathy, non arteritic§
C1_a52	Female	78	Polymyalgia rheumatica; Multisegmental osteochondrosis
C1_a53	Male	54	Polymyalgia rheumatica
C1_a54	Female	89	Migraine
C1_a55	Female	68	Bacterial endocarditis
C1_a56	Male	52	Unilateral facial hypaesthesia, psychosomatic genesis
C1_a57	Male	71	Isolated extracranial GCA
C1_a58	Female	74	Central retinal artery occlusion, embolic
C1_a59	Male	78	Anterior ischemic optic neuropathy, non arteritic§
C1_a60	Male	76	Anterior ischemic optic neuropathy, non arteritic
C1_a61	Female	54	Anterior ischemic optic neuropathy, non arteritic§

§Temporal artery biopsy performed, negative.

Table S2: Final clinical diagnoses other than cGCA in cohort 2 (n = 84).

**Patient**	**Sex**	**Age**	**Final clinical diagnosis**
C2_a01	Male	91	Central retinal artery occlusion, embolic
C2_a02	Female	79	Central retinal artery occlusion, embolic
C2_a03	Male	79	Central retinal artery occlusion, embolic
C2_a04	Male	75	Central retinal artery occlusion, embolic
C2_a05	Female	82	Combined branch retinal artery/vein occlusion, non arteritic
C2_a06	Female	77	Anterior ischemic optic neuropathy, non arteritic
C2_a07	Male	78	Anterior ischemic optic neuropathy, non arteritic
C2_a08	Female	79	Combined central retinal artery and vein occlusion, non arteritic
C2_a09	Female	85	Anterior ischemic optic neuropathy, non arteritic
C2_a10	Female	81	Anterior ischemic optic neuropathy, non arteritic
C2_a11	Female	86	Central retinal artery occlusion, embolic
C2_a12	Male	79	Branch retinal artery occlusion, non arteritic
C2_a13	Female	81	Branch retinal artery occlusion, embolic
C2_a14	Female	76	Anterior ischemic optic neuropathy, non arteritic§
C2_a15	Male	73	Branch retinal artery occlusion, embolic
C2_a16	Female	58	Anterior ischemic optic neuropathy, non arteritic§
C2_a17	Female	73	Branch retinal artery occlusion, embolic
C2_a18	Female	61	Anterior ischemic optic neuropathy, non arteritic§
C2_a19	Male	62	Anterior ischemic optic neuropathy, non arteritic
C2_a20	Male	64	Central retinal artery occlusion, embolic
C2_a21	Female	66	Anterior ischemic optic neuropathy, non arteritic§
C2_a22	Female	90	Central retinal artery occlusion, embolic
C2_a23	Female	71	Branch retinal artery occlusion, embolic
C2_a24	Male	70	Branch retinal artery occlusion, embolic
C2_a25	Female	84	Branch retinal artery occlusion, embolic
C2_a26	Male	84	Central retinal artery occlusion, embolic
C2_a27	Male	79	Central retinal artery occlusion, embolic
C2_a28	Male	81	Central retinal artery occlusion, embolic
C2_a29	Male	52	Central retinal artery occlusion, embolic
C2_a30	Female	64	Central retinal artery occlusion, embolic
C2_a31	Male	72	Central retinal artery occlusion, embolic§
C2_a32	Female	73	Central retinal artery occlusion, embolic
C2_a33	Male	71	Central retinal artery occlusion, embolic
C2_a34	Male	56	Branch retinal artery occlusion, embolic

C2_a35	Male	77	Anterior ischemic optic neuropathy, non arteritic
C2_a36	Male	80	Branch retinal artery occlusion, embolic
C2_a37	Female	83	Central retinal artery occlusion, embolic
C2_a38	Male	71	Central retinal artery occlusion, embolic
C2_a39	Female	79	Branch retinal artery occlusion, embolic
C2_a40	Male	89	Central retinal artery occlusion, embolic[§]
C2_a41	Male	59	Anterior ischemic optic neuropathy, non arteritic
C2_a42	Female	60	Branch retinal artery occlusion, embolic
C2_a43	Female	61	Hemicentral retinal artery occlusion, embolic
C2_a44	Male	59	Central retinal artery occlusion, embolic
C2_a45	Male	70	Branch retinal artery occlusion, embolic
C2_a46	Female	63	Central retinal artery occlusion, embolic
C2_a47	Male	77	Central retinal artery occlusion, embolic
C2_a48	Female	88	Central retinal artery occlusion, embolic
C2_a49	Male	71	Anterior ischemic optic neuropathy, non arteritic (Waldenström macroglobulinemia)
C2_a50	Male	72	Anterior ischemic optic neuropathy, non arteritic
C2_a51	Male	89	Central retinal artery occlusion, embolic
C2_a52	Male	76	Branch retinal artery occlusion, embolic
C2_a53	Female	80	Central retinal artery occlusion, embolic
C2_a54	Male	79	Branch retinal artery occlusion, embolic
C2_a55	Male	58	Anterior ischemic optic neuropathy, non arteritic
C2_a56	Male	67	Branch retinal artery occlusion, embolic
C2_a57	Female	75	Central retinal artery occlusion, embolic
C2_a58	Male	78	Hemicentral retinal artery occlusion, embolic
C2_a59	Female	71	Anterior ischemic optic neuropathy, non arteritic
C2_a60	Male	59	Anterior ischemic optic neuropathy, non arteritic
C2_a61	Female	62	Central retinal artery occlusion, embolic
C2_a62	Male	52	Anterior ischemic optic neuropathy, non arteritic
C2_a63	Female	78	Central retinal artery occlusion, embolic
C2_a64	Female	89	Central retinal artery occlusion, embolic
C2_a65	Male	55	Anterior ischemic optic neuropathy, non arteritic
C2_a66	Male	56	Combined cilioretinal artery and central retinal vein occlusion, non arteritic
C2_a67	Female	68	Branch retinal artery occlusion, embolic
C2_a68	Male	74	Anterior ischemic optic neuropathy, non arteritic[§]
C2_a69	Male	53	Anterior ischemic optic neuropathy, non arteritic
C2_a70	Female	90	Branch retinal artery occlusion, embolic
C2_a71	Female	89	Branch retinal artery occlusion, embolic
C2_a72	Female	82	Central retinal artery occlusion, embolic
C2_a73	Male	75	Central retinal artery occlusion, embolic
C2_a74	Male	87	Anterior ischemic optic neuropathy, non arteritic
C2_a75	Female	72	Branch retinal artery occlusion, embolic
C2_a76	Male	79	Central retinal artery occlusion, embolic
C2_a77	Female	82	Anterior ischemic optic neuropathy, non arteritic[§]
C2_a78	Male	67	Branch retinal artery occlusion, embolic
C2_a79	Male	94	Hemicentral retinal artery occlusion, embolic
C2_a80	Male	72	Central retinal artery occlusion, embolic
C2_a81	Female	73	Anterior ischemic optic neuropathy, non arteritic
C2_a82	Male	75	Anterior ischemic optic neuropathy, non arteritic[§]
C2_a83	Male	56	Anterior ischemic optic neuropathy, non arteritic
C2_a84	Female	84	Anterior ischemic optic neuropathy, non arteritic

[§]Temporal artery biopsy performed, negative.
